# Einfluss der Coronapandemie auf Schmerzpatienten: Welche Auswirkungen der Pandemie auf ihre Versorgung nehmen Patienten mit chronischen Schmerzen wahr?

**DOI:** 10.1007/s00482-021-00549-2

**Published:** 2021-04-22

**Authors:** Thomas H. Cegla, Astrid Magner

**Affiliations:** 1 Schmerzklinik Wuppertal, Helios Universitätsklinikum Wuppertal, Klinik Bergisch Land, Im Saalscheid 5, 42369 Wuppertal, Deutschland; 2 Helios Universitätsklinikum Wuppertal, Universität Witten/Herdecke, Heusnerstraße 40, 42283 Wuppertal, Deutschland; 3 Schmerzklinik Wuppertal, Helios Universitätsklinikum Wuppertal, Universität Witten/Herdecke, Klinik Bergisch-Land GmbH, Im Saalscheid 5, 42369 Wuppertal, Deutschland

**Keywords:** Coronavirus, Pandemie, Chronischer Schmerz, Multimodal stationäre Therapie, Biopsychosoziales Krankheitsmodell, Coronavirus, Pandemic, Chronic pain, Multimodal inpatient treatment, Biopsychosocial disease model

## Abstract

**Hintergrund:**

Erkrankungen verursacht durch das neuartige Coronavirus (SARS-CoV-2) haben in einem sehr dynamischen Verlauf zu einer Pandemie geführt. Die epidemiologische Lage von nationaler Tragweite erforderte Infektionsschutzmaßnahmen mit dem Ziel, die Morbidität und Mortalität zu senken. Eine Überbelastung der Gesundheitssysteme sollte vermieden werden. Die Maßnahmen zur Bekämpfung der Pandemie hatten und haben Auswirkungen auf das öffentliche und private Leben. Chronisch schmerzkranke Patienten sind ebenfalls betroffen.

**Fragestellung:**

Welche Auswirkungen der Pandemie auf ihre Versorgung nehmen Patienten mit chronischen Schmerzen wahr?

**Methode:**

Siebzig Schmerzpatienten nach einer multimodal stationären Therapie wurden in einem standardisierten Interview telefonisch zu ihrem Befinden wie folgt befragt: Führten die Coronabedingungen zu einer Zunahme der Schmerzbelastung, verschlechterte sich die Stimmung und hatte die Pandemie einen nachteiligen Einfluss auf die schmerzmedizinische Versorgungssituation?

**Ergebnisse:**

Die Veränderungen im biopsychosozialen Bereich sind für die Patienten erlebbar und wirken sich auf die Gesamtsituation aus. Chronisch schmerzkranke Patienten waren vom sogenannten Lockdown betroffen. Patienten bringen eine Verschlechterung von Stimmung und ihrer Schmerzsituation zu einem großen Anteil mit den coronabedingten Maßnahmen in Verbindung. Deutlich ist die Stimmungsverschlechterung (70 % der Befragten). Eine Schmerzzunahme wird bei 44 % der Befragten mit den durch die Pandemie verursachten Veränderungen in Zusammenhang gebracht. 39 % der Patienten geben eine Verschlechterung ihrer schmerzmedizinischen Versorgung an. Die Maßnahmen zur Bekämpfung der Pandemie hatten und haben Auswirkungen auf das Leben chronisch kranker Schmerzpatienten.

**Diskussion:**

Die bestehenden, auch im Vergleich zur Situation ohne Pandemie begrenzten Möglichkeiten einer koordinierten Versorgung von Schmerzpatienten im Anschluss an einen multimodal stationären Aufenthalt auch in schwierigen Situationen beizubehalten, ist notwendig. Die negativen Auswirkungen einer Reduktion medizinischer Versorgung sind ein Argument für eine multimodal ambulante Weiterversorgung insbesondere nach erfolgter stationärer Behandlung.

## Abstract (en)

**Background:**

Diseases caused by the novel coronavirus (SARS-CoV-2) have led to a pandemic in a very dynamic manner. The epidemiological situation of national importance required infection control measures with the aim of reducing morbidity and mortality. An overburdening of the healthcare system should be avoided. The measures taken to combat the pandemic have had an impact on public and private life. Patients suffering from chronic pain have also been greatly affected.

**Question:**

Which impact on their care do patients with chronic pain experience?

**Method:**

After multimodal inpatient treatment 70 pain patients were interviewed by telephone in a standardized fashion. They were asked about their condition as follows: did the changes due to the pandemic result in an increase in pain levels, a deterioration in mood and did the pandemic have a negative impact on the supply of pain medication?

**Results:**

Changes in the biopsychosocial area were experienced by the patients and affected their overall well-being. Chronically ill pain patients were particularly affected by the lockdown. A large number of patients associated a deterioration in mood and aggravation of the chronic pain with measures resulting from the pandemic. The mood deterioration was clearly associated (70% of respondents). An increase in pain was associated with changes caused by the pandemic in 44% of respondents. Of the patients 39% reported a deterioration of the pain management. The measures taken to combat the pandemic have had and still have an impact on the lives of chronically ill pain patients.

**Discussion:**

It is necessary to maintain the limited but still existing options of coordinated care for pain patients following a multimodal inpatient stay, even in difficult situations. The negative effects of a reduction in medical care are an argument in favor of multimodal outpatient care, especially after inpatient treatment.

## Hintergrund und Fragestellung

### Die Versorgungssituation chronisch schmerzkranker Patienten unter den weitgehenden Einschränkungen für das öffentliche Leben

Am 25. März wurde vom Bundestag eine „epidemiologische Lage von nationaler Tragweite“ gesehen und am 27. März die gesetzliche Grundlage für weitreichende Maßnahmen mit dem Gesetz zum Schutz der Bevölkerung bei einer epidemiologischen Lage von nationaler Tragkraft gelegt. Bund und Länder vereinbarten von Mitte März bis Anfang Mai 2020 weitgehende Einschränkungen für das öffentliche Leben [4, 6, 8, 18]. Die Maßnahmen zur Bekämpfung der Pandemie hatten und haben Auswirkungen auf das öffentliche und private Leben. Im Gesundheitsbereich wurden z. B. elektive Aufnahmen in die Kliniken verschoben [18]. Nach Egger haben Veränderungen im biopsychosozialen Bereich bei chronisch kranken Schmerzpatienten krankheitsmodellierende Auswirkungen [10]. Führen die unmittelbaren Folgen der Pandemie demnach zu erkrankungsrelevanten Konsequenzen chronischer Schmerzpatienten?

Fallen Therapien aus, ohne dass eine genügende Eigenkompetenz gebildet wurde, kann sich dies auf die Gesamtsituation auswirken. Dies betrifft physiotherapeutische und psychologische Verfahren. Ebenso negativ ist der Wegfall sozialer Kontakte. Erzwungene Veränderungen der Pharmakotherapie durch Versorgungsengpässe [9], bedingt durch Lieferschwierigkeiten aus den ebenfalls von der Pandemie betroffenen asiatischen Ländern (Beispiel Antidepressivaumstellung), wirken sich potenzierend negativ aus. Die Gesamtheit dieser Veränderungen beschreibt die Auswirkung der im Rahmen der Pandemie getroffenen Maßnahmen in Bezug auf die Versorgung chronisch kranker Schmerzpatienten. Mobilitätsabnahme, Reduktion sozialer Kontakte und eine verringerte Inanspruchnahme medizinischer Versorgungsstrukturen führen zu wahrnehmbaren Veränderungen [2, 5, 8, 14, 18]. Ausgehend von dieser These erfolgte eine standardisierte telefonische Befragung von zuvor (Juni bis Dezember 2019) stationär multimodal behandelten chronisch schmerzkranken Patienten, die vom sogenannten Lockdown betroffen waren.

## Studiendesign und Untersuchungsmethoden

Die Studie unserer schmerztherapeutischen Klinik wurde als prospektives, deskriptives Verfahren zur Erfassung von Häufigkeitsverteilungen und Mittelwerten (Standardabweichungen) per mündlicher Telefonabfrage konzipiert.

Nach positivem Votum der Ethikkommission (Universität Witten/Herdecke, Antrag Nr. 122/2020) und der Zustimmung des Datenschutzbeauftragten des Helios Klinikums Wuppertal wurde eine Stichprobe aus 147 Schmerzpatienten ausgewählt und nach schriftlich erfolgter Zustimmung in der Zeitspanne vom 28.07.2020 bis zum 09.09.2020 befragt. Alle Patienten waren zuvor im Zeitraum von Juni bis Dezember 2019 in der eigenen schmerztherapeutischen Klinik stationär multimodal behandelt worden. Es wurden nur Patienten inkludiert, deren Aufnahme mehr als ein halbes Jahr zurücklag, um einen ausreichenden zeitlichen Abstand zu der SARS2-Pandemie zu gewährleisten.

Patienten mit schriftlicher Einverständniserklärung wurden in einem standardisierten Interview von einer approbierten Psychotherapeutin der Abteilung nach den gängigen Kriterien der Durchführungsobjektivität telefonisch befragt. Neben den Angaben des Interviews flossen das Alter in Lebensjahren sowie das Geschlecht der Probanden in die Untersuchung ein. Weitere persönliche Daten wurden nicht erhoben.

### Telefoninterview

Das Interview sah vor, drei relevante Bereiche zu erheben. Auf einer numerischen Intervallskala von 0 bis 10 wurde die quantitative Ausprägung erfasst (Tab. 1):

- Besteht eine Zunahme der Schmerzbelastung infolge der Coronabedingungen? Falls ja: Wie hoch ist der negative Einfluss auf einer Skala von 0 bis 10?
- Führt die coronageprägte allgemeine Lebenssituation zu einer Verschlechterung der Stimmung? Bei positivem Votum: Wie groß ist der negative Einfluss auf einer Skala von 0 bis 10?
- Hat die Pandemie einen nachteiligen Einfluss auf die schmerzmedizinische Versorgungssituation? Im positiven Fall: Welche Ausprägung hat der negative Einfluss auf einer Skala von 0 bis 10?

**Table 1:** 

Das Coronavirus ist derzeit in aller Munde und wir leben nun schon längere Zeit mit den damit verbundenen Einschränkungen. Wir würden Sie gerne fragen, ob die bestehende Situation sich auch aktuell auf Ihre Schmerzsituation auswirkt:										
1. Führt die gegenwärtige Situation zu einer Zunahme ihrer Schmerzbelastung?										
☐ Ja										
☐ Nein										
Wie stufen Sie den negativen Einfluss auf einer Skala von 0 bis 10 ein?										
0	1	2	3	4	5	6	7	8	9	10
2. Führt die gegenwärtige Situation zu einer Verschlechterung Ihrer Stimmung/Ihres Wohlbefindens?										
☐ Ja										
☐ Nein										
Wie stufen Sie den negativen Einfluss auf einer Skala von 0 bis 10 ein?										
0	1	2	3	4	5	6	7	8	9	10
3. Hat die Pandemie gegenwärtig einen nachteiligen Einfluss auf Ihre schmerzmedizinische Versorgungssituation?										
☐ Ja										
☐ Nein										
Wie stufen Sie den negativen Einfluss auf einer Skala von 0 bis 10 ein?										
0	1	2	3	4	5	6	7	8	9	10

Die statistische Auswertung erfolgte mit der Tabellenkalkulation Excel. Zur Unterscheidung zwischen coronabedingt belasteten und nichtbelasteten Personen wurden die Daten dichotomisiert und geschlechtergetrennt ausgewertet. Auf eine altersbezogene Ermittlung der Häufigkeiten wurde verzichtet, da sich die Stichprobe über eine zu geringe Altersrange erstreckt.

Die Frage nach der schmerzmedizinischen Versorgungssituation wurde allgemein gehalten. Ziel war es herauszufinden, ob Schmerzpatienten eine Verschlechterung ihrer Versorgungssituation empfinden. Eine Differenzierung der unterschiedlichen Versorgungsarten ließ sich nicht statistisch auswerten.

## Ergebnisse

Insgesamt wurden von 147 angefragten chronische Schmerzpatienten nach stationär multimodaler Behandlung 70 Patienten mit positivem Rücklauf interviewt. 91 % (*N* 63) der Teilnehmenden waren über 50 Jahre alt. 63 % (*N* 44) der Patienten waren weiblich, 37 % (*N* 26) waren männlich.

Die deutlichste coronabedingte Belastungsfolge ergab sich bei der Frage nach der Stimmungsverschlechterung mit einer Zustimmung von 70 % (*N* 49). Hierunter lag der Anteil betroffener Männer mit 73 % (*N* 19) knapp über dem Anteil der Frauen mit 68 % (*N* 30). In der Gesamtstichprobe lag die mittlere Höhe des negativen Einflusses der Coronabedingungen auf die Stimmung auf der Rating-Skala von 0 bis 10 bei 6,5 (M = 6,5; SD = 2,0) wobei die Frauen (M = 6,7; SD = 1,9) den mittleren Wert der Männer (M = 6,3; SD = 2,01) leicht übertrafen.

Eine Zunahme der Schmerzbelastung unter Coronabedingungen wurde von 44 % (*N* 31) der Studienteilnehmer angegeben. 50 % der weiblichen (*N* 22) und 35 % (*N* 9) der männlichen Befragten beantworten die Frage nach den verschlechternden Auswirkungen auf die Schmerzbelastung mit einem Gesamtmittelwert von 6,3 (M = 6,3; SD = 2,1) positiv. Hierunter ergab sich für Frauen der Wert von 6,6 (M = 6,6; SD = 2,1) und für Männer von 5,6 (M = 5,6; SD = 1,9; Tab. 2 und 3).

**Table 2:** 

	Schmerz % (*N*)	Stimmung % (*N*)	Versorgung % (*N*)
*Gesamt*	44 (31)	70 (49)	39 (27)
*Weiblich*	50 (22)	68 (30)	39 (17)
*Männlich*	35 (9)	73 (19)	39 (10)

**Table 3:** 

	Schmerz	Stimmung	Versorgung
*Gesamt*	6,3 (SD = 2,1)	6,5 (SD = 2,0)	5,7 (SD = 1,8)

Eine nachteilige Wirkung der Pandemiezeit auf die schmerzmedizinische Versorgungssituation gaben 39 % (*N* 27) an, worunter 39 % (*N* 10) der Männer und 39 % (*N* 17) der Frauen positiv mit einem Gesamtmittelwert von 5,7 (M = 5,7; SD = 1,8) votierten. Der Mittelwert der männlichen und weiblichen Befragten lag jeweils bei 5,7 (m: M = 5,7; SD = 1,6; w: M = 5,7; SD = 1,9).

## Diskussion

### Erkrankungen durch Coronavirus (SARS-CoV-2) – eine epidemiologische Lage von nationaler Tragkraft

Erkrankungen verursacht durch das neuartige Coronavirus (SARS-CoV-2) haben von China ausgehend weitere Länder erreicht. Die sehr dynamische Situation hat zu einer Pandemie geführt. Die Infektionsschutzmaßnahmen wurden angepasst mit dem Ziel, die Morbidität und Mortalität zu senken. Eine Überbelastung der Gesundheitssysteme sollte vermieden werden.

### Die Versorgungssituation chronisch schmerzkranker Patienten

Die Pandemie hatte und hat Auswirkungen auf die Versorgungssituation chronisch schmerzkranker Patienten, am ehesten durch die Maßnahmen zur Pandemiebekämpfung mit ihren sozialen Einschränkungen. Chronische Schmerzen sind häufig und betreffen diverse individuelle und gesellschaftliche Lebensbereiche. Die Prävalenz chronischer Schmerz in Deutschland beträgt rund 17 % und variiert je nach Ursache [20]. Das biopsychosoziale Krankheitsmodell trägt dieser Erkenntnis Rechnung und ist die Grundlage für das heutige Verständnis einer multimodalen berufsgruppenübergreifenden Diagnostik und Therapie chronischer Schmerzen [10]. Eine Überlegenheit einer mehrdimensionalen Schmerztherapie gegenüber eindimensionalen Behandlungsansätzen besteht [1, 15]. Bei höherer Chronifizierung ist deren Effektivität von der Therapiedauer und -intensität abhängig [14]. Im ambulanten Bereich wird diese Art der koordinierten Versorgung gefordert, ist aber bisher abhängig von Pilotprojekten und dem persönlichen Engagement. Interdisziplinäre berufsgruppenübergreifende Schmerzkonferenzen sind eine Möglichkeit des Austauschs und der Koordination [7]. Als Grundlage der interdisziplinären Zusammenarbeit kann auf ein systemtheoretisches Verständnis von Gesundheit und Krankheit zurückgegriffen werden. Jedes Verhalten steht danach in einem Netz oder Gefüge von Bezugsgrößen, die sich gegenseitig beeinflussen [10, 13]. In der praktischen Erfahrung haben wir aber in unseren ambulanten Schmerztherapien (Klinikambulanz, MVZ) und stationären Einrichtungen weniger Patienten behandelt. Physiotherapeutische Maßnahmen standen nicht wie gewohnt zur Verfügung. Die Verordnung von Medikamenten war nicht immer problemlos möglich. Lieferschwierigkeiten gab es z. B. bei Doxepin. Die bestehende Versorgungsstruktur ist bezogen auf die Anschlussbehandlung nach einer multimodalen Schmerztherapie unbefriedigend. Auch dies erleben wir im klinischen Alltag. Stationäre Behandlungserfolge sind nur dann nachhaltig, wenn die multimodalen Ansätze auch ambulant weiterverfolgt werden können. Eine Versorgung, die vor der Pandemie unzureichend war, wird unter Pandemiebedingungen nicht besser.

### Veränderungen im biopsychosozialen Bereich unter weitgehenden Einschränkungen für das öffentliche Leben

Die Veränderungen im biopsychosozialen Bereich sind für die Patienten erlebbar und wirken sich auf die Gesamtsituation aus [3, 5, 11, 12, 17, 20]. Chronisch schmerzkranke Patienten waren vom sogenannten Lockdown betroffen. Patienten bringen eine Verschlechterung der Stimmung (zu 44 %) und ihrer Schmerzsituation (zu 70 %) zu einem großen Anteil mit den coronabedingten Maßnahmen in Verbindung. Deutlich ist die Stimmungsverschlechterung. Vergleichszahlen der Stimmungsverschlechterung der gesunden Bevölkerungspopulation liegen nicht vor, sodass die absoluten Werte nur eine eingeschränkte Aussagekraft haben. Eine negative Auswirkung auf das Erkrankungsgeschehen ist jedoch wahrscheinlich und für die Folgen von Quarantänemaßnahmen bei schwerwiegenden Coronaausbrüchen belegt [16]. Eine Schmerzzunahme wird bei 44 % der Befragten mit den durch die Pandemie verursachten Veränderungen in Zusammenhang gebracht. Es zeigt sich ein deutlicher Zusammenhang zwischen sozialem Geschehen und Schmerz. Mobilitätsveränderung, geringere soziale Kontakte sowie seltenere oder keine Besuche speziell schmerzmedizinischer Einrichtungen könnten eine Rolle spielen. Der letzte Punkt wird bei 39 % als nachteilig empfunden. Die bestehenden begrenzten Möglichkeiten, eine koordinierte Versorgung von Schmerzpatienten im Anschluss an einen multimodal stationären Aufenthalt auch in schwierigen Situationen beizubehalten, ist notwendig. Die negativen Auswirkungen einer Reduktion medizinischer Versorgung sind ein Argument für eine multimodal ambulante Weiterversorgung, insbesondere nach erfolgter stationärer Behandlung und dies nicht nur in dieser besonderen Zeit einer Pandemie. Dass Schmerz auch ein soziales Phänomen ist, zeigt sich durch die Auswirkungen, die ein verändertes Umfeld hat. Die sozialen Hintergründe und Lebensweisen der Patienten sind genauso zu berücksichtigen wie allgemeine gesellschaftliche Veränderungen [13, 19]. In der Pandemie, am ehesten ausgelöst durch die Maßnahmen zur Bekämpfung der Pandemie, berichten die chronisch kranken Schmerzpatienten über deutliche Einschränkungen im Befinden und ihrer schmerztherapeutischen Versorgung. Zur genauen Differenzierung sind weitere Untersuchungen notwendig.

## Fazit für die Praxis

- Die Coronapandemie nimmt negativen Einfluss auf das körperliche und seelische Wohlbefinden sowie die ambulante schmerzmedizinische Versorgungssituation chronisch schmerzerkrankter Patienten.
- Ein nachhaltiger positiver Effekt einer multimodalen stationären Schmerztherapie macht eine ambulante Weiterbehandlung notwendig, die alle Ebenen des biopsychosozialen Modells berücksichtigt.
- Allgemeine gesellschaftliche Veränderungen müssen in der Behandlung chronisch kranker Schmerzpatienten stärker berücksichtigt werden.
- Im (pandemischen) Krisenmanagement sollte die psychosoziale Gesundheit chronisch erkrankter Schmerzpatienten ein integraler Bestandteil sein.
- In Zeiten kollektiver Ausnahmezustände erhöhen sich die Versorgungsaufwendungen des Gesundheitssystems inklusive einzelner Fachgruppen. Dies inkludiert das schmerztherapeutische multimodale Setting mit Medizinern und Psychotherapeuten in besonderer Weise.
- Auch für chronische Schmerzpatienten bedeutet die Coronapandemie eine erhebliche psychosoziale Zusatzbelastung und eine Verminderung ausgleichender Kompensationsmöglichkeiten. Dies zieht besondere Zusatz- und Anpassungsleistungen der Behandler aller Ebenen nach sich.
